# Social Capital and Technological Literacy in Taiwan

**DOI:** 10.1100/2012/149132

**Published:** 2012-04-19

**Authors:** Hsieh-Hua Yang, Fen Fen Huang, Yi-Horng Lai, Hung-Jen Yang, Jui-Chen Yu

**Affiliations:** ^1^Department of Health Care Administration, Oriental Institute of Technology, No. 58, Section 2, Szechwan Road, Banciao District, New Taipei City 220, Taiwan; ^2^Department of Industrial Technology Education, National Kaohsiung Normal University, No. 62, Shenjhong Road, Yanchao Township, Kaohsiung County 824, Taiwan; ^3^Division of Collection and Research, National Science and Technology Museum, No. 720, Jiouru 1st Road, Kaohsiung 807, Taiwan

## Abstract

The burgeoning interest in social capital within the technology community represents a welcome move towards a concern for the social elements of technological adaptation and capacity. Since technology plays an ever larger role in our daily life, it is necessary to articulate social capital and its relationship to technological literacy. A nationwide data was collected by area sampling, and position generator was used to measure social capital. Regression model was constructed for technological literacy. Age, gender, education, income, web access, and social capital were included as independent variables. The results show that age, gender, education, web access, and social capital were good predictors of technological literacy. It is concluded that social capital is helpful in coping with rapid technological change. Theoretical and empirical implications and future research are discussed.

## 1. Introduction

Technology plays an ever larger role in our daily life. The range of technology available today is very broad, as how to meet human's needs, to live healthier, more productive lives. Obviously, technology is not divorced from its social and cultural context, but little attention has been paid on technological literacy and social factors. The burgeoning interest in social capital within the technology community represents a welcome move towards a concern for the social elements of technological adaptation and capacity. Social capital theory posits that the extent and effectiveness of social and community relations modifies the returns to human capital [1, 2]. Technology is created from human interaction.

Social dimensions are claimed to have been central in the creation of knowledge and behavior. The process of transferring technological literacy requires greater attention to the relational dimension of social capital. In this study, the aim is to articulate social capital and its relationship to technological literacy.

## 2. Literature Review

### 2.1. Technological Literacy

There is wide agreement that technological literacy should be defined very broadly. Hayden [3] defined technical literacy as having the knowledge and ability to select, properly apply, then monitor, and evaluate appropriate technology given the context. The abilities are needed for consumers and citizens to make decisions and think broadly across disciplines [4]. According to ITEA, technological literacy is the “ability to use, manage, assess, and understand technology” [5]. Frank [6] concluded that developing technological literacy refers to the following dimensions: acquiring technological multidisciplinary knowledge, experiencing synthesis and engineering design processes, becoming familiar with engineering, using a top-down approach, performing cost/benefit analyses, and becoming familiar with the concept of engineering systems thinking, and with principles of project management. Humans need more than just knowledge of current technology and skills in using it; they also need additional abilities to accommodate and use the new and changing technologies of the future.

Wonacott [7] gave two reasons why technological literacy should be defined very broadly. One is the broad range of human problems that technology might solve; the other is that the creation of new technologies and extension of old technologies will increase the ranges of both. The standards of technological literacy are grouped in 5 categories [8]. One of these categories is “Technology and Society” which includes cultural, social, economic, and political effects of technology; role of society in the development and use of technology. Apparently, the technological literacy is considered to be related to society.

### 2.2. Technological Literacy and Social Factors

Young et al. [9] indicated that a technologically literate person would possess a number of general characteristics. One of the characteristics was recognizing that society shapes technology as much as technology shapes society. They thought that the values and culture of society affect technology, and technological development sometimes favors the values of certain groups more than others. They also argued that such development traditionally has favored the values of males more than those of females. However, Zuzovsky [10] found it gender-free in the attainment of technological literacy among sixth graders in Israel. Young et al. [9] also posited that a technologically literate person would be prepared to take part in public forums and make their opinions heard on issues involving technology. Rutherford [11] thought that the technologies are not merely tools but products of the culture that reflect social values. Toscano [12] encouraged the discussions on technological literacy not to emphasize solely skills training, but to broaden the focus on technology by analyzing the social, political, and historical aspects of technologies.

Empirical findings support that social factor plays a critical role in technological fields. Sahay and Robey [13] revealed strikingly different patterns of implementation and social consequences from the same technology and demonstrated the role of social interpretations in the implementation of information technology. Chiu et al. [14] found that social capital would influence individuals' knowledge sharing in virtual communities. Mallett [15] indicated that the active participants interact continuously throughout the adopting process are effective in eliciting social acceptance of renewable energy innovations. Bridgland and Whitehead [16] examined information literacy and indicated that social capital is an important factor for the sustainability of effective information literacy programs.

In science education, Bybee [17] reminded us that some individuals advocated a science-technology society or S-T-S theme for school programs. Such an approach emphasized the pervasive nature of technology in our society. Educational intervention can improve performance in the area of perception of technological literacy [18]. Judson [19] identified the link existed between gains in technology literacy and achievement in the areas of reading, mathematics, and language arts. However, Fanta-Vagenshtein and Chen [20] found that illiteracy did not preclude the development of knowledge in general, technological knowledge. Their view seems contradictive, but the pervasive nature in our society makes it possible.

Many studies have used micro- and macroperspective on the study of social capital. The focus has been on the level of individual social capital and creation of social capital in countries, states, or organizations. Reich and Kaarst-Brown [21] showed that social capital led to an increase in intellectual capital and the organizational advantage achieved. Chou [22] posited that social capital impacts growth by assisting in the accumulation of human capital, by affecting financial development through its effects on collective trust and social norms, and by facilitating networking between firms that result in the creation and diffusion of business and technological innovations. Sherif et al. [23] posited that knowledge management system would positively affect an organization's ability to build social capital and that social capital would enhance a firm's ability to create and transfer knowledge. Yli-Renko et al. [24] suggested that fostering social capital within the firm and in external relationships would significantly benefit the firm's knowledge base and international growth.

Collins and Hitt [25] posited that effectively managing existing tacit knowledge stocks and transferring knowledge. They explain how firms use relational capabilities to build relational capital with partners. In turn, relational capital facilitates the transfer of tacit knowledge between collaborating partners.

### 2.3. Social Capital

The sources of social capital may trace to Hawe and Shiell [26]. Portes [27] suggests that social capital means the ability to secure benefits through membership in networks and other social structures. Nahapiet and Ghoshal [28] defined social capital as the sum of the actual and potential resources embedded within, available through, and derived from the network of relationships possessed by individual or social units. A broad view by Burt [29] defined social capital as an asset embedded in relationships of individuals, communities, networks, or societies. In general, sociologists elaborated three dimensions of an individual's social capital: structural capital, relational capital, and cognitive capital. The structural dimension describes the network itself; the relational dimension emphasizes the ties that bind the network together, and the cognitive dimension focuses on the content of the social capital. Lin's theory [30] grounded in the classic tradition of capital theories can elaborate the three dimensions thoroughly. It defines social capital as “resources embedded in a social structure which are accessed and/or mobilized in purposive actions” [30]. Thus, social capital contains three elements: resources embedded in a social structure; accessibility to such social resources by individuals; use or mobilization of such social resources by individuals in purposive actions.

The social capital theory [30] has specifically proposed that access to and use of social resources embedded in social networks can have two types of outcomes, instrumental and expressive returns. For instrumental action, there are three possible returns: economic, political, and social. For expressive action, social capital is a means to consolidate resources and to defend against possible resource losses.

There are two methodologies used to measure access to social capital, name generators, and position generators. Position generator was proposed by Lin and Dumin [31]. This measurement samples positions in a hierarchical structure, rather than sampling ego-centered interpersonal ties, to the extent that social capital reflects embedded resources in the structure, then this approach should yield meaningful information regarding ego's access to such structurally embedded resources. From the responses, it becomes possible to construct three indicators. Range is the distance between the highest and lowest accessed positions, and it represents the accessibility to different hierarchical positions in the society. Extensity is the number of positions accessed, and it indicates the heterogeneity of accessibility to different positions. Upper reachability indicates the prestige or status of the highest position accessed.

 The exploration of the relationship between the social capital and technological literacy will signify the importance of interpersonal dynamics involved in the creation of technological literacy.

## 3. Method

### 3.1. Participants

The sampling frame was nationwide and composed of a stratified (by administrative district) probability sample of over 15 years old persons including outlying islands and mountain townships. The over-all criterion that should be applied in choosing a sampling design is to so design the sample that it will yield the desired information with the reliability required at a minimum cost or, conversely, that at a fixed cost, it will yield estimates of the statistics desired with the maximum reliability possible [32].

 Because a complete frame of reference was not available, area sampling method was adopted. An area sampling is a method in which the area to be sampled is subdivided into smaller blocks which are selected at random and then subsampled or fully surveyed. The entire 1100 sample came from 17 counties, 7 cities, and 1 island. And it is a reasonable random sample of the population of Taiwan. There were 2 respondents who did not complete the questionnaire.

### 3.2. Measurement

A validated instrument developed by Xu [33] was applied to measure technological literacy. This instrument includes transportation, media, architecture, manufacture, and synthesized dimensions. Each dimension has 9 questions of multiple choices. These 5 dimensions construct technologic literacy battery with reliability of 0.70–0.85.

The questionnaire for generating social capital was adopted from Lin et al. [34]. The respondents were asked “among your relatives, friends, or acquaintances, are there people who have the following jobs?” Following the questions were fifteen “job” positions sampled from two structural dimensions: occupational prestige and class. Position-generated variables are summarized in Table 1.

 Three indexes were constructed from the position-generator items, extensity, upper reachability, and range. These three measures of position data were highly correlated, and a composite variable was constructed. A factor score, as Table 2, was computed for both male and female respondents as a weighed sum of the three measures (0.02 extensity + 0.50 range + 0.51 upper reachability). Both range and upper reachability carried much more weight than extensity. This composite variable was social capital in this study.

 Gender, age, income, and web access were also included in the questionnaire. The measurement of income was classified into 9 degrees. Below, 4,999 is the 1st degree, 5,000–9,999 is the 2nd degree, 10,000–14,999 is the 3rd degree, 15,000–19,999 is the 4th degree, 20,000–39,999 is the 5th degree, 40,000–59,999 is the 6th degree, 60,000–79,999 is the 7th degree, 80,000–99,999 is the 8th degree, and over 100,000 is the 9th degree. The participants were asked if they search information by browsing internet as the measurement of web access.

## 4. Results

As in Table 3, the participants were 569 females and 529 males with average age of 35. More than 62% of them were graduated from college, 28.4% from high school, and the rest from senior high school. Most of the participants searched information by browsing internet.

 The mean score of technological literacy is 32.27. The highest score is 42. It means no one get full score. For transportation, media, architecture, manufacture, and synthesized dimensions, the mean scores are shown in Table 3. The scores of transportation and synthesized dimensions are more than 7, and media and architecture dimensions are less than 6. The average degree of their income was 5.42. It meant that the mean of income was more than NTD 40,000 per month. The mean of social capital was 55.03.

 As in Table 4, the regression model shows that age, education, web access, and social capital are significant factors of technological literacy, but not income. *R* square is 0.185. Technological literacy is increased by age. Higher educated people have higher technological literacy. Those who can search information on web have higher technological literacy. And people with higher score of social capital have more advantage on technological literacy than the other.

Table 5 shows regression models for transportation, media, architecture, manufacture, and synthesized dimensions of technological literacy. Most of the models have similar pattern. *R* square for each dimension is between 0.094 and 0.152. Gender is not a significant factor for most of the models. But for synthesized dimension, females have more advantage than males. Technological literacy is different among three levels of education, but for architecture and synthesized dimensions, the junior high graduates have not less advantage than senior high graduates.

## 5. Discussion and Conclusion

The aim of this study was to articulate the social capital and its relationship to technological literacy. A nationwide data was collected, and position generator was used to measure social capital. The results supported the applicability of social capital theory. Technological literacy will be increased by age, education, web access, and social capital. It supports the S-T-S theme that emphasized the pervasive nature of technology in our society.

 In the knowledge society, knowing how to use technology is increasingly important, whether we are looking for a job, marketing a service, or shopping for a product. We are also expected to be able to use other devices, like microwaves, ovens for cooking, computers, e-mail for communication, motorbikes, and cars for transportation, that become part of everyday life at home, at work, or in the community. Technology is used to solve human problems, meet human needs, and help human living conveniently and easily. In short, society shapes technology as much as technology shapes society [9].

### 5.1. The Mechanism of Social Capital

The premise behind the notion of social capital is rather simple and straightforward: investment in social relations with expected returns in the marketplace [30]. The market may be economic, political, labor, or community that is consistent with the standards of technological literacy offered by ITEA.

The results showed that the return of different social capital was positively correlated with technological literacy. The mechanisms that embedded resources in social networks were proposed by Lin et al. [35]. For one, it facilitates the flow of information. The information communication and sharing offered by social capital has contribution on the interpretations of technology. Further, the different social interpretations of the same technology will exert different patterns of implementation [13]. The social capital is important for the acceptance of new technology and the sustainability of renewable innovations. Second, these social ties may exert influence on the actors. Some social relations, due to their prestige and positions, also carry more resources and exercise greater power of influence. The means of reachability for both male and female respondents were over 67 which were rather high in the occupation hierarchy. Those social relations would have great influence on the respondents and reinforce their attitude and behavior about technology. Third, social resources may be conceived as certifications of the individual's social credentials. Having higher reachability means that someone has good social relations, and it will broaden one's vision about technology. And the last social relations are expected to reinforce identity and recognition. Being assured of one's worthiness as a member of a social group, the feeling of belongingness will increase their sharing of the same value and norm about technological literacy.

It is concluded that the social capital theory is applicable for explaining technological literacy.

### 5.2. Position-Generator Is Used to Explore the Details

There are two methodologies commonly used to measure access to social capital: name generators and position generators. The name generator is the more common methodology and has been used extensively in the network literature [36–40]. The general technique is to pose one or more questions about the ego's contacts in certain contexts or situations. In studies [36–40], the students were asked to nominate 3 to 5 best friends.

 Name generator tends to be bound with specified content areas, to elicit stronger rather than weaker ties, and to locate access to individuals rather than social positions. Lin et al. [35] argued that name generators fall short on some issues important to the development of social capital as a theory and proposed the position generators. The position generators use a sample of ordered structural positions salient in a society (occupations, authorities, work units, class, or sector) and ask respondents to indicate contacts in each of the positions. From the responses, it becomes possible to construct measures of range of accessibility, extensity, and upper reachability.

 The compositions of social capital in this study and in the earlier study [34] are different. In this study, social capital was composed of (0.02 extensity + 0.50 range + 0.51 upper reachability). The extensity variable carried the least weight, and two other variables were almost equally weighted. While in the earlier research [34], social capital was composed of (0.15 extensity + 0.65 range + 0.21 upper reachability). The range variable carried three times more weight than the other two variables. Obviously, the importance of upper reachability variable in social capital had been increased during these years. The reason may be due to different study design, subjects, or context. It needs further research.

 During the latter half of the 20th century in Taiwan, a rapid expansion of education for all but particularly for women occurred along with the rapid social and economic changes. In this study, 62.8% of the respondents were graduated from university. In the study [34], only 24.0% of the respondents were graduated from college or more. Obviously, the education had been rapidly expanded during these 10 more years. Since education is benefit in accessibility, the rapid expansion of Taiwan's education will have effect on social capital. However, within these three important variables, extensity, upper reachability, and range, only the range variable was increased. But, gender differences was decreased during these years after comparing with the result of the earlier research [34]. There were no significant differences of reachability, extensity, and range between males and females. Yet, females still play the role of keeping household well-being. They were more likely to access nurses, while males were more likely to access assemblymen/women, reporter, owner of small factory/firm, electrician, and truck driver, but not nurses. van Emmerik [41] indicated that hard social capital refers to accumulated task-oriented resources that can be used to achieve valued career outcomes, and soft social capital refers to emotional support resources that can be used to achieve socioemotional support. It could be inferred that the different accessibility between males and females also might be due to occupation segregation. Further research can explore the inference.

### 5.3. Enlightenment from Synthesized Technological Literacy

The social capital theory [30] has specifically proposed that access to social resources can have two types of outcomes, instrumental and expressive returns. It is reasonable to suppose that men and women may specialize in the creation of different types of social capital. This can be used to explain the difference between females and males of synthesized dimension of technological literacy.

 The synthesized dimension of technological literacy includes items of environmental protection, future technology trends, and populations. Three reasons can be applied to explain the gender difference of synthesized dimension. First, as van Emmerik [41] stated, hard social capital develops from instrumental ties that arise in the course of work role performance and involve the exchange of job-related resources. And soft social capital refers to emotional support resources that can be used to achieve socioemotional support and involve the exchange of friendship and social support. Females are expected to hold more emotional-responsive attitudes and be more sensitive to the human problems, environmental pollution. Second, females are more likely to have soft social capital by mobilizing emotional support resources. The soft social capital will reinforce their concern for the environment and human well-being through information, influence, and control. The last, as B. Weber and C. Weber [42] extend social capital theory by integrating conative fit and affective fit into relational fit, and proved relational fit would facilitate knowledge transfer and creation. The soft social capital of women is relational fit for transferring synthesized knowledge of technology.

 There is still some gap between the results and Emmerik's finding. van Emmerik's study [41] indicated that men were more effective in creating hard social capital, but women were not found to be the emotional specialists they often are thought to be. While in our results, women still play the role of keeping household well-being. They are more caring and create more soft social capital. We infer the gap due to cultural difference, and it needs further research.

### 5.4. Further Reflection

People holding the model of technological determinism believe that a technology, once created, takes on a life of its own. The result seems to move to a contrary view, social determinism. It is suggested that a third model is needed to address both of these propensities in conjunction with the technology and society.

Notwithstanding, social capital is helpful in accommodating and coping with rapid and continuous technological change. The social interaction within community provides forum [43] for generating creative and innovative solutions for technological problems, acting through technological knowledge both effectively and efficiently. And a technological literate society has the ability to assess technology and its involvement with the human world judiciously. Empirical research tested that social determinism has its limitation. Chou et al. [44] found that social capital might be a double-edged sword that is both a resource and a burden in studying IT outsourcing. Also, Johnson [45] provided a framework for consideration of technologies that are frequently viewed as either a source of power or frustration. In conclusion, technology shapes society as much as society shapes technology.

## Figures and Tables

**Table 1:** Summary of position-generated variables.

Variables	Mean or percent			Gender significance
	Sample	Males	Females	
Extensity	6.60	6.82	6.40	0.066
Upper reachability	66.79	66.32	67.23	0.386
Range of prestige	41.67	41.39	41.93	0.601
Accessed positions				
Physician (78)	53.1%	51.8%	54.3%	0.432
Lawyer (73)	26.5	26.1	26.9	0.785
Owner of large factory/firm (70)	25.5	28.0	23.2	0.072
Assemblymen/women (69)	23.6	27.0	20.4	0.010
Manger of large factory/firm (62)	36.6	38.4	35.0	0.259
High school teachers (60)	50.1	52.4	48.0	0.148
Division head (55)	29.2	30.6	27.9	0.353
Reporter (55)	21.4	24.4	18.6	0.022
Nurse (54)	65.6	57.5	73.1	0.000
Owner of small factory/firm (48)	52.9	57.3	48.9	0.005
Police (40)	54.4	54.1	54.7	0.856
Electrician (36)	55.8	61.8	50.3	0.000
Truck driver (31)	37.6	45.0	30.8	0.000
Office workman/guard (26)	82.7	83.7	81.7	0.381
Housemaid, cleaning worker (22)	45.2	43.9	46.4	0.430

**Table 2:** Factor structures of access to social capital.

	Sample (*N* = 1098)	Male (*N* = 529)	Female (*N* = 569)
Factor eigenvalues			
I	2.42	2.46	2.39
II	0.19	0.20	0.18
III	0.39	0.35	0.43
Factor loading			
Extensity	0.73	0.75	0.71
Range	0.97	0.97	0.97
Upper reachability	0.97	0.98	0.97
Factor scoring			
Extensity	0.02	0.02	0.02
Range	0.50	0.48	0.51
Upper reachability	0.52	0.53	0.51

**Table 3:** Description of participants.

Variables	*N*	%
Gender		
Female	569	51.8
Male	529	48.2
Education		
Junior	96	8.7
Senior	312	28.4
College	690	62.8
Web access		
Yes	800	72.9
No	298	27.1

*Variables *	*Mean*	*SD*

Technological literacy (5–42)	32.27	7.11
Transportation dimension (0–9)	7.19	1.88
Media dimension (0–9)	5.64	1.69
Architecture dimension (0–9)	5.76	1.47
Manufacture dimension (0–9)	6.55	1.71
Synthesized dimension (0–9)	7.18	1.82
Age (18–85)	35.25	12.58
Income (1–10)	5.42	2.28
Social capital (0–68.08)	55.03	17.06

**Table 4:** Factors influencing technological literacy.

Variables	*B*	s.e.	*P*
Constant	17.117	1.318	0.000
Age	0.118	0.019	0.000
Gender (female/male)	0.481	0.394	0.223
Education			
Senior/junior high	2.019	0.817	0.014
College/junior high	4.929	0.832	0.000
Income (1–9)	0.051	0.099	0.606
Web accessibility (yes/no)	3.032	0.498	0.000
Social capital	0.074	0.012	0.000
*R*	0.430		
*R* square	0.185		

**Table 5:** Factors for 5 dimensions of technological literacy.

Variables	Transportation *B* (s.e.)	Media *B* (s.e.)	Architecture *B* (s.e.)	Manufacture *B* (s.e.)	Synthesized *B* (s.e.)
Constant	3.664 (0.356)***	3.034 (0.325)***	3.655 (0.288)***	3.424 (0.327)***	3.555 (0.345)***
Age	0.025 (0.005)***	0.021 (0.005)***	0.029 (0.004)***	0.020 (0.005)***	0.023 (0.005)***
Gender (female/male)	0.157 (0.106)	−0.070 (0.097)	−0.066 (0.086)	0.147 (0.098)	0.301 (0.103)**
Education					
Senior/junior high	0.486 (0.220)*	0.413 (0.201)*	0.163 (0.178)	0.589 (0.203)**	0.329 (0.213)
College/junior high	1.203 (0.224)***	1.046 (0.205)***	0.508 (0.182)**	1.120 (0.206)***	1.003 (0.217)***
Income (1–9)	0.003 (0.027)	−0.007 (0.024)	0.011 (0.022)	−0.005 (0.024)	0.039 (0.026)
Web access (Y/N)	0.718 (0.134)***	0.609 (0.123)***	0.336 (0.109)**	0.650 (0.124)***	0.642 (0.130)***
Social capital	0.017 (0.003)***	0.014 (0.003)***	0.010 (0.003)***	0.016 (0.003)***	0.017 (0.003)***
*R*	0.383	0.355	0.306	0.364	0.390
*R* square	0.147	0.126	0.094	0.132	0.152

*P < 0.05,  **P < 0.01,  ***P < 0.001.
